# Association between Proximity of the Elementary School and Depression in Japanese Older Adults: A Cross-Sectional Study from the JAGES 2016 Survey

**DOI:** 10.3390/ijerph18020500

**Published:** 2021-01-09

**Authors:** Megumi Nishida, Masamichi Hanazato, Chie Koga, Katsunori Kondo

**Affiliations:** 1 Advanced Preventive Medical Sciences, Graduate School of Medical and Pharmaceutical Sciences, Chiba University, 1-8-1 Inohana, Chuo-ku, Chiba-shi, Chiba 260-8672, Japan; 2 Takenaka Corporation, 1-13, 4-chome, Hommachi, Chuo-ku, Osaka 541-0053, Japan; 3 Center for Preventive Medical Sciences, Chiba University, 1-33 Yayoi-cyo, Inage-ku, Chiba-shi, Chiba 263-8522, Japan; hanazato@chiba-u.jp (M.H.); chiekoga@chiba-u.jp (C.K.); kkondo@chiba-u.jp (K.K.); 4 Center for Gerontology and Social Science, National Center for Geriatrics and Gerontology, 7-430 Morikoka-cho, Obu-shi, Aichi 474-8511, Japan

**Keywords:** older adults, depression, neighborhood environment, elementary school proximity, intergenerational exchange, age-friendly cities, Japan

## Abstract

Depression among older adults is one of the most critical public health issues. The proximity of elementary schools has been positively associated with neighborhood social cohesion and quality of life. However, no studies have identified an association between the proximity of elementary school and older adults’ mental health. Therefore, this study aimed to examine the association between the proximity of elementary schools, one of the core facilities of neighborhood communities in Japan, and depression in older adults. A total of 131,871 participants (63,430 men 73.7 ± 6.1 years, 68,441 women 73.8 ± 6.2 years) were analyzed from the Japan Gerontological Evaluation Study (JAGES) 2016 survey. Logistic regression analysis showed that there was no association between distance to elementary school and depression among males. However, among females, compared with the participants living within 400 m from the nearest elementary school, the odds ratio of depression for those living between 400 and 799 m and more than 800 m away were 1.06 (95% confidence interval (CI) 1.00–1.12) and 1.07 (95% CI 1.00–1.15), respectively. The findings may be useful when considering the design of communities around elementary schools and the planning of facilities as a population-based approach to promote mental health of older women.

## 1. Introduction

The incidence of depression is increasing worldwide, and a World Health Organization (WHO) report estimated that more than 300 million people, equivalent to 4.4% of the world’s population, had depression in 2017. The total population with depression increased by 18.4% between 2005 and 2015 [1], and its prevalence is exceptionally high among older adults [1]. The age-standardized incidence of depression worldwide is also increasing in regions with higher sociodemographic indicators [2]. Depression significantly reduces the quality of life and increases the risk of suicide, dementia, functional decline, elder abuse, and disability in older adults [3,4,5,6,7], and constitutes a significant public health issue.

In 2006, the WHO advocated for primordial prevention by recommending a change of attitudes, behaviors, and social values, which can be achieved by encouraging positive health behaviors, preventing the adoption of risk behaviors, eliminating established risk behaviors, and promoting the concept of health as a social good [8]. Primordial prevention requires a population-based approach, which targets the whole population irrespective of the risk level, and this is different from the high-risk approach, which focuses only on individuals or population groups with the highest risk of disease. In addition, it must confront the social, cultural, and community aspects of increased risk. Moreover, population-based approaches achieve considerably greater health gains than high-risk approaches due to the larger number of people involved [9].

These trends have intensified research on neighborhood environments that improve health [9,10]. There are many reports on the relationship between neighborhood environment and physical health, such as the impact of parks, sidewalks, or other built environments on physical activity and obesity rates [11,12]. There have also been studies on how the neighborhood environment affects mental health in older adults [13]. For example, the neighborhood built environment comprising high walkability, better access to transportation and destinations, better street connectivity, traffic safety, large amount of greenspace, and the social environment, such as high local social capital, is possibly related to improved mental health in older adults [14,15,16,17,18,19].

To develop a healthy neighborhood environment, the WHO has published the age-friendly cities guide that promotes active aging of older adults [20]. This guideline has indicators such as the accessibility of destinations, traffic safety, and social inclusion, and recommends the use of community facilities such as schools and recreation centers by all community members, including older adults, to help them participate in community activities and intergenerational exchange [21]. This implies that elementary schools can be considered components of age-friendly cities.

However, the relationship between elementary schools and the health of older adults has not been sufficiently studied. For example, the proximity to an elementary school, which represents the distance between the elementary school and the place of residence, has been shown to enhance neighborhood social cohesion as a place for increased interaction and group activities, improved quality of life, and increased children’s physical activity [22,23,24,25,26]. These participants may encompass the entire population of residents, including older adults and children. Furthermore, none of the previous studies examined the relationship between proximity to an elementary school and older adults’ health, particularly depression.

Therefore, we hypothesized that proximity to an elementary school might be associated with improved mental health of older adults. In Japan, elementary schools are one of the core facilities of a neighborhood community and are often used for local events, such as elections and athletic events, which may enhance social capital that is said to be associated with the health of individual or community health. In addition, the vicinity of the elementary schools may influence the safety of elementary school children on their way to and from school. Furthermore, people residing near elementary schools have many potential opportunities for daily contact with children, such as seeing, hearing, and talking to elementary school students on their way to and from school, which may promote intergenerational exchange. The purpose of this study was to examine the relationship between proximity to elementary schools and depression among Japanese older adults.

## 2. Methods

### 2.1. Population and Settings

This study used cross-sectional data from the Japanese Gerontological Evaluation Study (JAGES) conducted in 2016 [27]. JAGES is an epidemiological research-based project conducted every 3 years from 2010 and focuses on older adults from different regions across Japan. It aims to build scientific evidence on the role of preventive medicine in a healthy aging society. Participants were older adults in a community (age ≥65 years) who did not have physical or cognitive disabilities and had not received a certification of long-term care [27,28]. Thirty-nine municipalities in 19 prefectures across Japan participated in this research project. The survey was a self-administered questionnaire that was returned by postal mail from October 3 to December 5, 2016. The questionnaire forms were distributed to participants via the local government. Although the local governments were not randomly selected, they covered a wide range of characteristics in terms of region and population size in Japan. The questionnaire examined health status, psychological status, social support, social participation, hobbies, frequency of outings, life habitats, and demographic information, including sex, age, education level, equivalent income (annual household income), marital status, living arrangement, self-reported socioeconomic status, etc. A total of 180,021 data points (response rate: 70.2%) were identified in 2016. We excluded older adults who did not live independently with regards to their daily living based on their response to the following questions evaluating independence of activities of daily living (ADLs): “Do you regularly receive care and assistance for walking, bathing, toileting, etc.?”; the responses included (1) does not need care or assistance, (2) needs care or assistance but does not receive it, and (3) needs care or assistance but does receive it. We defined (3) as being dependent for activities of daily living [29]. A total of 131,871 individuals without missing data for the outcome (i.e., depression) and residential information for the explanatory variables were included in the analysis.

### 2.2. Outcome Variable

The outcome variable was depression. A short version of the Geriatric Depression Scale (GDS-15) was used to identify depression, which was indicated by a GDS-15 score of 5 or more in this study [30,31].

### 2.3. Explanatory Variable

The explanatory variable was the distance from the respondents’ place of residence to the nearest elementary school. For accuracy, we used the latitude and longitude of a representative point in the city-level location reference information provided by the Ministry of Land, Infrastructure, Transport and Tourism, rather than the respondent’s residence address. The network distance from the representative point of the respondent’s residence to the nearest elementary school was calculated using ArcGIS ver. 10.3.1 (Esri, Redlands, CA, USA) [32]. The network distances were categorized into four groups for every 400 m (<400, 400–799, 800–1199, and ≥1200 m). The 400-m category was determined on the basis of prior literature and guidelines on the proximity to the destinations [33,34,35]. Walking distance within five minutes of walking time was often represented by a radius measuring 400 m among urban planners [36]. The neighborhood concept proposed by Clarence Stein also suggests that elementary schools should be located at the center of neighborhood units within a 400-m walking distance of all residents [37].

### 2.4. Covariates

According to the covariates that were evaluated in previous studies, we used basic demographic information, including sex (male/female), age (65–69, 70–74, 75–79, 80–84, or ≥85 years), education level (≤9, ≥10 years, or missing), equivalent income (low: ≤1.9; middle: 2.0–3.9; high: ≥4 million yen/year; and missing), marital status (married, unmarried, others, missing), employment status (employed, unemployed, or missing), and family structure (living alone, living with a family member, others (e.g., living in institutional care or missing)) as covariates [38,39,40]. Furthermore, we added the frequency of outings (>4 times a week, 1–3 times a week, 1–3 times a month, less than a few times a year, or missing), driving habits (no driving or driving), and frequency of meeting friends (>4 times a week, 1–3 times a week, 1–3 times a month, less than a few times a year, or missing) as covariates as these were previously reported to be associated with depression [41,42]. House-owned status (owned, rented or otherwise, or missing) and duration of residence (<20, 20, 20–39, 40–59, 60–79, ≥80 years, and missing) were added as variables that could be assumed as confounders of residence and depression [43,44]. To adjust for urbanity, the township population density was added to the covariate as quintiles [45,46].

### 2.5. Statistical Analysis

Descriptive statistics were used to summarize the participants’ characteristics, their number, and the sex-stratified percentage of subjects for each variable. Chi-square tests were used to report sex-related differences for each variable. Logistic regression analysis was performed to investigate the association between depression and accessibility to the elementary school. Odds ratios (ORs) and 95% confidence intervals (CIs) were calculated by sex because of the possibility of sex-related differences in descriptive statistics. Stata 16.1/IC (StataCorp, College Station, TX, USA) was used for all statistical analyses.

## 3. Results

Table 1 shows the sex-stratified results of the descriptive statistics of the 131,871 individuals. The incidence of depression was higher in males than in females (22.9% and 22.3%, respectively, *p* < 0.001). Among the older adults, 15.2% lived near an elementary school (<400 m), and there were no sex-related differences in the proximity to an elementary school. The percentage of the target population of females (51.9%) was higher than that of males (48.1%). The results of the chi-square test showed that a higher proportion of males had more than 10 years of education (70.5% of male participants and 64.4% of female participants, *p* < 0.001) and employment (31.4% male participants and 20.1% female participants, *p* < 0.001). Moreover, men were more likely to be married (84.8% of males and 61.6% of females, *p* < 0.001) and less likely to live alone (37.9% of males and 48.9% of females, *p* < 0.001). A higher percentage of male participants drove cars than female participants (76.1% and 40.1%, respectively, *p* < 0.001). Regarding psychosocial factors, female participants tended to meet with friends more frequently than the male participants (more than once a week: 48.9% and 37.9%, respectively, *p* < 0.001).

The OR and 95% CI for the association between living near an elementary school and depression are shown in Table 2. Among male participants, there was no significant association between distance to the elementary school and the incidence of depression. However, among female participants, compared with the participants who lived less than 400 m away from the nearest elementary school, those who lived 400–799, 800–1199, and >1200 m away were 1.06 (95% CI 1.00–1.12), 1.07 (95% CI 1.00–1.14), and 1.07 (95% CI 1.00–1.15) times, respectively, more likely to have depression. In terms of the association between covariates and depression, the risk of depression was higher for both men and women with lower education and income, no job, unmarried, living alone, not living in an owned house, and no driving habits. Less frequent going out and seeing friends was associated with a higher risk of depression for both men and women, but this trend was more pronounced in women than in men. Considering age group as a covariate, older age was associated with a lower risk of depression for both men and women aged 70 years and older compared to those aged 65–69 years. The risk of depression has been reported to increase with age, although this trend was reversed when educational history, income, employment, and marital status were included as covariates in this analysis [42,47]. Among the associations between covariates and depression, the trend for men and women differed in duration of residence. Men aged 60 years and older were at a higher risk of depression than those who lived for less than 20 years, but there was no association between duration of residence and depression in women.

## 4. Discussion

The present study confirmed the association between depression and basic demographic information as reported in previous studies: Frequency of going out and visiting friends, home ownership, length of residence in a community, and population density. For example, a significant association was found between higher education and higher income and lower risk of depression. Being married, having a family, and having a job were also associated with a lower risk of depression. Further, those who went out more frequently and had frequent interactions with friends had a lower risk of depression. Regarding the residential environment, the results suggest that owning a house and having a longer residence duration may be protective factors for the risk of depression in men. Although the association between population density and risk of depression was been consistent, the present study showed an association between higher population density and lower risk of depression [14,48].

The present analysis also suggests that older women who live near an elementary school may have a lower risk of depression than those who do not, even after controlling for basic demographic information such as frequency of going out, frequency of meeting friends, household status, duration of residence, and population density, which are reported to be associated with depression [31,32,33,34,35,36,37,38,39]. The ORs for the association between elementary school proximity and depression were smaller than those of basic demographic information and other data; however, the large number of participants analyzed in this study allowed us to detect the effect. We discuss several mechanisms by which the environment near an elementary school may reduce the risk of depression among older women.

### 4.1. High Social Capital as the Center of the Community in the Elementary School Neighborhood Environment

Social capital may be related to the association between high proximity to an elementary school and low depression. Putnam defined social capital as “features of social organization, such as networks, norms, and social trust that facilitate coordination and cooperation for mutual benefit” [49]. In addition, evidence suggests that areas with higher social capital, that is, more connected people and more cooperative behavior, have lower rates of anxiety, depression, and other psychiatric disorders. A study examining the relationship between public space and sense of community in Australian neighborhoods reported a significant association between distance to the nearest school and sense of community, with greater distance to the nearest school being linked with a lower sense of community [50]. The authors considered that participation in more neighborhood and school activities may contribute to expanding social networks, developing friendships, and a greater sense of community. In contrast, an American study reported no linear association between the distance to a school and social cohesion, which is one index of social capital [51]. The lack of association was assumed to be related to the means of transportation for getting to school. If a school has many students who commute to school by bus or private transportation, there may be fewer opportunities to increase the school’s collective effects. In other words, students are more likely to walk to school in their neighborhood up to a certain threshold distance, and there is a positive relationship between social cohesion and school proximity up to that threshold. However, the relationship between social cohesion and school proximity may disappear farther away from that threshold [51]. Thus, we can speculate that the association between elementary school proximity and social capital is related to whether the school is within walking distance. More than 90% of Japanese elementary school students walk to school [52]. This study found a higher odds ratio for depression among female older adults living at a distance of more than 400 m from an elementary school, which roughly supports the findings of previous studies.

Moreover, the Ministry of Health, Labour and Welfare suggests that elementary schools can be a base for social capital because they are a venue for student activities and a place for interaction between parents and community members. Moreover, it cites the fact that the activities of community organizations are often organized within the elementary school district as a possible reason for the above factors [53]. A questionnaire survey of Japanese adults older than 65 years reported that women were less likely to go out, work, and engage in hobby activities than men. However, in contrast, women were more likely to participate in neighborhood and community association activities [54]. Given that participation in elementary school-based activities of community organizations is relevant in the mechanism by which high elementary school proximity contributes to improved social capital, sex-related differences in community activity participation may explain the association, especially for women.

### 4.2. The Safety of the Elementary School Neighborhood Environment

The second factor regarding high proximity to elementary schools that was associated with lower depression among older adults can be assumed to be the high safety level in the environment near elementary schools. The Cabinet Office in Japan promotes traffic safety on school routes through the cooperation of residents, schools, boards of education, municipalities, and the police, which includes community-based supervision, police patrols, and traffic restrictions [55]. In Japan, school zones are designated as critical areas for traffic safety policies to protect children from traffic accidents and are located within a 500-m radius of an elementary school on the school route. The school zone imposes various traffic restrictions, such as installing new crosswalks and curb mirrors, sidewalk widening, one-way traffic and speed restrictions, and a ban on traffic during school hours [56]. These strategies have contributed to traffic-related safety in the elementary school neighborhood environment.

Perceived neighborhood traffic-related safety was negatively related to possible depression in older adults [57,58]. Furthermore, neighborhood risk perceptions are reported to be higher in women than in men [59]. Thus, women may be more sensitive and stressed about low traffic safety in their neighborhoods. The results of this study match those of earlier studies about the high incidence of depression in women living at a greater distance from elementary schools.

### 4.3. Potential for Contact and Intergenerational Interaction with Children in an Elementary School Neighborhood Environment

A third factor that may contribute to less depression as elementary school proximity increases is that more frequent contact with children in the elementary school neighborhood environment may foster generativity. Erikson defined the concept of generativity as an interest in contributing to the well-being of others, especially the younger generation, and as a developmental challenge in middle age and later years [60,61]. Childbearing, parenting, and social interactions possibly foster generativity by increasing interest in the younger generation’s development and well-being [60,62,63]. Furthermore, higher levels of generational interest and more frequent generational interactions, that is, greater generativity are associated with better mental health in old age [62,63,64,65]. According to the findings of a US study, intergenerational exchange was associated with improved subjective health status and psychological well-being [66]. In another study of older Japanese adults, positive intergenerational interaction was suggested to foster generativity and improve well-being in older age [61]. Previous research reported that generativity tends to be higher in women than in men [67]. Another study has shown that generativity impacts subjective well-being, with the trend being more robust in women than in men [62]. The results of this study are consistent with previous research findings, which showed that women tend to have greater generativity and better well-being.

### 4.4. Strengths and Limitations

The strength of this study is that it is one of the few studies that investigated the relationship between elementary school proximity and depression among older adults in a large epidemiological dataset covering both urban and rural areas. Understanding the relationship between depression in older adults and their neighborhood environment is essential for supporting community-dwelling older adults. Clarifying the health risk-preventing effects of elementary school neighborhoods, which exist in tens of thousands of locations nationwide, may have implications for city design, such as developing senior living centers near elementary schools.

Another strength of this analysis is that it uses objective data as an explanatory variable. Subjective indicators using questionnaires have often been used to analyze environmental factors; however, it has been noted that there are individual differences in the perception of subjectively measured environmental factors. The use of objective data as an indicator facilitates objective assessment and comparison of regional differences.

Nonetheless, this study has the following limitations. First, the cross-sectional design precludes the identification of a causal relationship. The second limitation is that respondents who needed long-term care and were not independent in their activities of daily living (ADLs) were excluded from this analysis. The results are only for community-dwelling older adults who can live independently before being certified for long-term care. The third limitation is that some of the residential data used in the analysis may differ from the actual address. For privacy reasons, the participant’s residence point data were substituted with the latitude and longitude of the representative points of the block-level location reference information provided by the Ministry of Land, Infrastructure, and Transport. Therefore, the network distance to the nearest elementary school, which is the explanatory variable in this study, differs from the actual distance from the point of residence. However, since the average radius at the block was 258.7 m, few respondents were expected to move across this explanatory variable’s 400-m category. Finally, we cannot precisely discern how elementary school proximity affects depression among older female respondents because our results did not specifically identify the factors in the elementary school neighborhood environment that prevent depression among older women. It is unknown whether it is the elementary school environment’s safety and walkability or influence by direct or indirect contact with children that contributes to the abovementioned effect. A more detailed analysis is needed in the future, as clarification of the mechanisms will allow more effective and specific interventions to be implemented.

Despite the limitations described above, this study presents findings that can help create a community that prevents depression among older adults.

## 5. Conclusions

This study suggests that proximity to an elementary school may be a protective factor for the mental health of community-dwelling older women living independently in the community, confirming part of our hypothesis. It may encourage participation in community activities based on the elementary school and promotes interaction with children, as promoted by the age-friendly cities guide [20]. In the future, clarifying the mechanism of the protective effect of elementary school proximity on depression among older women may help examine specific interventions to prevent depression. Furthermore, examining the mechanisms underlying the gender differences in this study may help develop effective approaches for both men and women. The above research to elucidate such mechanisms could contribute to a design guide for age-friendly cities.

## Figures and Tables

**Table 1:** Sex-stratified descriptive statistics for the total study population.

Characteristics		Male *n* = 63,430	Female *n* = 68,441	χ^2^ Value (df)
Depression, *n* (%)				7.3 (1) **
	Normal	48,896 (77.1%)	53,184 (77.7%)	
	Depression	14,534 (22.9%)	15,247 (22.3%)	
Distance to elementary school, m, *n* (%)				1.4 (3)
	<400	9533 (15.0%)	10,402 (15.2%)	
	400–799	21,491 (33.9%)	23,239 (34.0%)	
	800–1199	17,727 (27.9%)	18,955 (27.7%)	
	≥1200	14,679 (23.1%)	15,845 (23.2%)	
Population density, person/km^2^, *n* (%)				16.0 (3) **
	<1119	15,325 (24.2%)	16,934 (24.7%)	
	1119–4985	15,842 (25.0%)	17,200 (25.1%)	
	4986–9377	16,332 (25.7%)	17,000 (24.8%)	
	≥9378	15,931 (25.1%)	17,307 (25.3%)	
Age group, years, *n* (%)				11.9 (4) *
	65–69	20,322 (32.0%)	21,471 (31.4%)	
	70–74	17,206 (27.1%)	18,611 (27.2%)	
	75–79	13,869 (21.9%)	15,358 (22.4%)	
	80–84	8214 (12.9%)	8757 (12.8%)	
	≥85	3819 (6.0%)	4244 (6.2%)	
Education level, years, *n* (%)				584.7 (2) ***
	≤9	18,142 (28.6%)	23,378 (34.2%)	
	≥10	44,718 (70.5%)	44,081 (64.4%)	
	Missing	570 (0.9%)	982 (1.4%)	
Equivalent income, million yen/year, *n* (%)				1188.9 (3) ***
	Low (≤1.9)	24,660 (38.9%)	26,325 (38.5%)	
	Mid (2–3.9)	22,086 (34.8%)	20,242 (29.6%)	
	High (≥4)	6495 (10.2%)	6036 (8.8%)	
	Missing	10,189 (16.1%)	15,838 (23.1%)	
Marital status, *n* (%)				9297.9 (3) ***
	Married	53,790 (84.8%)	42,190 (61.6%)	
	Unmarried	8617 (13.6%)	25,086 (36.7%)	
	Others	457 (0.7%)	422 (0.6%)	
	Missing	566 (0.9%)	743 (1.1%)	
Work status, *n* (%)				3559.3 (2) ***
	Employed	19,901 (31.4%)	13,740 (20.1%)	
	Unemployed	37,341 (58.9%)	41,791 (61.1%)	
	Missing	6188 (9.8%)	12,910 (18.9%)	
Living arrangement, *n* (%)				2735.6 (3) ***
	Living alone	6247 (9.8%)	12,564 (18.4%)	
	With family members	48,700 (76.8%)	44,021 (64.3%)	
	Other facilities	5820 (9.2%)	7952 (11.6%)	
	Missing	2663 (4.2%)	3904 (5.7%)	
Household status, *n* (%)				91.1 (2) ***
	Owned	55,534 (87.6%)	58,987 (86.2%)	
	Rental/others	7464 (11.8%)	8715 (12.7%)	
	Missing	432 (0.7%)	739 (1.1%)	
Duration of residence, years, *n* (%)				4403.8 (5) ***
	<20	12,314 (19.4%)	13,174 (19.2%)	
	20–39	19,323 (30.5%)	19,698 (28.8%)	
	40–59	17,857 (28.2%)	28,011 (40.9%)	
	60–79	10,840 (17.1%)	5900 (8.6%)	
	≥80	2332 (3.7%)	733 (1.1%)	
	Missing	764 (1.2%)	925 (1.4%)	
Outgoing frequency, *n* (%)				70.0 (4) ***
	≥4 times/week	42,713 (67.3%)	44,935 (65.7%)	
	1–3 times/week	11,718 (18.5%)	13,249 (19.4%)	
	1–3 times/month	1529 (2.4%)	1352 (2.0%)	
	A few times/year	320 (0.5%)	250 (0.4%)	
	Missing	537 (0.8%)	481 (0.7%)	
Driving habit, *n* (%)				17,478.5 (1) ***
	No	15,170 (23.9%)	41,030 (59.9%)	
	Yes	48,260 (76.1%)	27,411 (40.1%)	
Frequency of meeting friends, *n* (%)				3544.5 (4) ***
	≥4 times/week	8474 (13.4%)	11,149 (16.3%)	
	1–3 times/week	15,554 (24.5%)	22,331 (32.6%)	
	1–3 times/month	12,727 (20.1%)	13,713 (20.0%)	
	A few times/year	19,227 (30.3%)	11,898 (17.4%)	
	Missing	835 (1.3%)	1176 (1.7%)	

*** *p* < 0.001, ** *p* < 0.01, * *p* < 0.05.

**Table 2:** The sex-stratified association between depression and the explanatory variables.

Characteristics	Male				Female			
	*n*	OR	95% CI	*p*-Value	*n*	OR	95% CI	*p*-Value
Distance to elementary school, m								
<400	9533	1.00			10,402	1.00		
400–799	21,491	1.01	0.95–1.07	0.773	23,239	1.06	1–1.12	0.059
800–1199	17,727	1.02	0.96–1.09	0.568	18,955	1.07	1–1.14	0.035
≥1200	14,679	1.00	0.93–1.07	0.923	15,845	1.07	1–1.15	0.051
Population density, person/km^2^								
<1119	15,325	1.00			16,934	1.00		
1119–4985	15,842	0.92	0.87–0.98	0.006	17,200	0.93	0.87–0.98	0.008
4986–9377	16,332	0.91	0.85–0.97	0.003	17,000	0.88	0.83–0.94	<0.001
≥9378	15,931	0.84	0.78–0.90	<0.001	17,307	0.87	0.81–0.93	<0.001
Age group, years								
65–69	20,322	1.00			21,471	1.00		
70–74	17,206	0.93	0.88–0.98	0.007	18,611	0.94	0.89–0.99	0.024
75–79	13,869	0.91	0.86–0.96	0.001	15,358	0.91	0.86–0.96	0.001
80–84	8214	0.95	0.88–1.01	0.117	8757	0.96	0.90–1.03	0.243
≥85	3819	0.87	0.79–0.95	0.003	4244	0.98	0.90–1.07	0.675
Education level, years								
≤9	18,142	1.00			23,378	1.00		
≥10	44,718	0.75	0.72–0.79	<0.001	44,081	0.80	0.77–0.83	<0.001
Missing	570	0.95	0.78–1.16	0.626	982	0.92	0.79–1.07	0.276
Equivalent income								
Low (≤1.9 million yen/year)	24,660	1.00			26,325	1.00		
Mid (2–3.9 million yen/year)	22,086	0.61	0.59–0.64	<0.001	20,242	0.65	0.62–0.68	<0.001
High (≥4 million yen/year)	6495	0.44	0.40–0.48	<0.001	6036	0.45	0.42–0.50	<0.001
Missing	10,189	0.87	0.82–0.92	<0.001	15,838	0.86	0.82–0.90	<0.001
Marital status								
Married	53,790	1.00			42,190	1.00		
Unmarried	8617	1.50	1.39–1.62	<0.001	25,086	1.18	1.12–1.24	<0.001
Others	457	1.50	1.22–1.84	<0.001	422	1.49	1.20–1.85	<0.001
Missing	566	1.44	1.20–1.74	<0.001	743	1.27	1.07–1.50	0.005
Work status								
Employed	19,901	1.00			13,740	1.00		
Unemployed	37,341	1.29	1.23–1.35	<0.001	41,791	1.21	1.15–1.28	<0.001
Missing	6188	1.36	1.26–1.46	<0.001	12,910	1.24	1.16–1.32	<0.001
Living arrangement								
Living alone	6247	1.00			12,564	1.00		
With family members	48,700	0.63	0.58–0.69	<0.001	44,021	0.82	0.77–0.87	<0.001
Others	5820	0.66	0.59–0.73	<0.001	7952	0.88	0.81–0.94	0.001
Missing	2663	0.70	0.62–0.79	<0.001	3904	0.88	0.80–0.96	0.006
Household status								
Owned	55,534	1.00			58,987	1.00		
Rental/others	7464	1.62	1.52–1.73	<0.001	8715	1.53	1.44–1.62	<0.001
Missing	432	1.27	1.02–1.58	0.032	739	1.33	1.12–1.56	0.001
Duration of residence, years								
≤19	12,314	1.00			13,174	1.00		
20–39	19,323	0.98	0.93–1.04	0.572	19,698	0.98	0.93–1.04	0.515
40–59	17,857	0.95	0.90–1.01	0.127	28,011	0.97	0.92–1.03	0.335
60–70	10,840	1.09	1.02–1.17	0.015	5900	0.97	0.89–1.05	0.483
≥80	2332	1.18	1.05–1.33	0.007	733	1.08	0.90–1.29	0.413
Missing	764	1.26	1.06–1.49	0.009	925	1.16	0.99–1.35	0.068
Outgoing frequency								
≥4 times/week	46,463	1.00			49,337	1.00		
1–3 times/week	13,684	1.53	1.46–1.60	<0.001	15,933	1.64	1.57–1.71	<0.001
1–3 times/month	2103	2.05	1.86–2.26	<0.001	2047	2.50	2.27–2.75	<0.001
A few times/year	555	3.10	2.59–3.70	<0.001	503	3.52	2.92–4.24	<0.001
Missing	625	1.26	1.04–1.51	0.017	621	1.29	1.07–1.56	0.008
Driving habit								
No	15,170	1.00			41,030	1.00		
Yes	48,260	0.77	0.73–0.81	<0.001	27,411	0.91	0.87–0.95	<0.001
Frequency of meeting friends								
≥4 times/week	9197	1.00			12,261	1.00		
1–3 times/week	17,061	1.26	1.17–1.36	<0.001	24,962	1.43	1.34–1.52	<0.001
1–3 times/month	14,100	1.61	1.49–1.74	<0.001	15,428	1.88	1.75–2.01	<0.001
A few times/year	22,005	2.53	2.35–2.71	<0.001	14,247	2.91	2.72–3.11	<0.001
Missing	1067	1.84	1.58–2.15	<0.001	1543	1.93	1.70–2.20	<0.001

OR: Odds ratios; CI: Confidence interval.

## Data Availability

Data is not suitable for public deposition due to ethical concerns. Data are from the JAGES study. Requests for data may be sent to the data management committee: dataadmin@jages.net.
